# Characterization and Genome Sequence of the Mycobacteriophage Donny

**DOI:** 10.1128/MRA.00373-20

**Published:** 2020-07-23

**Authors:** Lauren E. Colston, Miriam Segura-Totten, Ryan A. Shanks

**Affiliations:** a Department of Kinesiology, University of North Georgia, Dahlonega, Georgia, USA; b Department of Biology, University of North Georgia, Dahlonega, Georgia, USA; Queens College

## Abstract

We report the discovery of the novel bacteriophage Donny, a *Siphoviridae* virus that infects Mycobacterium smegmatis mc^2^155. Donny has a genome length of 69,691 bp and a G+C content of 68.5%. Donny shares 99% and 93% nucleotide identity with bacteriophages Acadian and Baee, respectively.

## ANNOUNCEMENT

The increasing discovery of mycobacteriophages provides further information on viral diversity, viral conservation, and the possible benefits of bacteriophage therapy (1, 2). The Science Education Alliance Phage Hunters Advancing Genomics and Evolutionary Science (SEA-PHAGES) program is designed for undergraduate students to discover and characterize novel bacteriophages that can add to the growing database (3). Mycobacteriophage Donny was isolated as part of the SEA-PHAGES program from an enrichment of a 0.22-μm-filtered soil sample (coordinates 34.527008N, 83.986983W) using the host Mycobacterium smegmatis mc^2^155. Infection with Donny results in plaques that are 0.1 cm in diameter, are clear in the center, and have smooth edges (4). Transmission electron microscopy (TEM) images indicate a *Siphoviridae* virus whose tail length has a striated pattern (4).

A Wizard DNA extraction kit (Promega) was used to isolate genomic DNA from Donny. To prepare a sequencing library from genomic DNA, a NEBNext Ultra II FS kit with dual-indexed barcoding was used. Default settings were used for all software unless otherwise specified. This library and those from 47 other phages were pooled and run on an Illumina MiSeq instrument. This run yielded 1,200,000 single-end 150-base reads from the Donny library. The raw reads were assembled using Newbler v2.9. When assembled, these reads provided 2,404-fold coverage of the Donny genome. The raw reads were assembled using Newbler v2.9 with default settings. The resulting single-phage contig was checked for completeness, accuracy, and phage genomic termini using Consed v29 as previously described (5). Donny contains a circularly permuted genome of 69,691 bp and a G+C content of 68.5% (6).

The Donny genome was annotated using Glimmer v3.02 (7), GeneMark v2.5p (8), BLAST (9), HHpred v3.0beta (10), and Phamerator (11). The E value cutoff used for BLASTP and HHpred was 10e^−4^. Donny was classified as a cluster B, subcluster B5 phage using nucleotide sequence similarity and shared gene content analysis as described in reference 12. The predicted number of genes is 97, with 26 having an assigned function. The Donny genome contained no genes coding for tRNAs, which is common in cluster B phages (13). Functions assigned to specific genes include structural proteins, terminase, and lysin A and B proteins. Genes 1 to 49 are predicted to be transcribed in the forward direction except for genes 7 and 8 and 42 to 46. Genes 50 to 97 are predicted to be transcribed in the reverse direction except for genes 67 to 77.

A nucleotide BLAST search demonstrated that Donny is closely conserved with subcluster B5 phages Acadian (99.99% identity) and Baee (93% identity) (14–16). Genes 1 to 20 are closely conserved with Baee in function and length. However, gene 21 is transcribed in the reverse direction in both Acadian and Donny, but not in Baee. Furthermore, gene 71 is unique to Donny and may represent a deletion of a longer gene, since it is shorter than average bacteriophage genes.

### Data availability.

The DNA sequence of Donny has been deposited in GenBank with accession no. MK524490. The sequencing reads are part of the Sequence Read Archive with SRA accession no. SRR10438086 under BioProject accession no. PRJNA488469.
